# Supplementary material for: Assessing the metabolic and physiological costs of oviparity in the epaulette shark (Hemiscyllium ocellatum)
Source: Biol Open. 2025 Nov 5;14(11):bio062076. doi: 10.1242/bio.062076 (PMC12641477; doi:10.1242/bio.062076)
Supplement: Supplementary information [file biolopen-14-062076-s1.pdf]

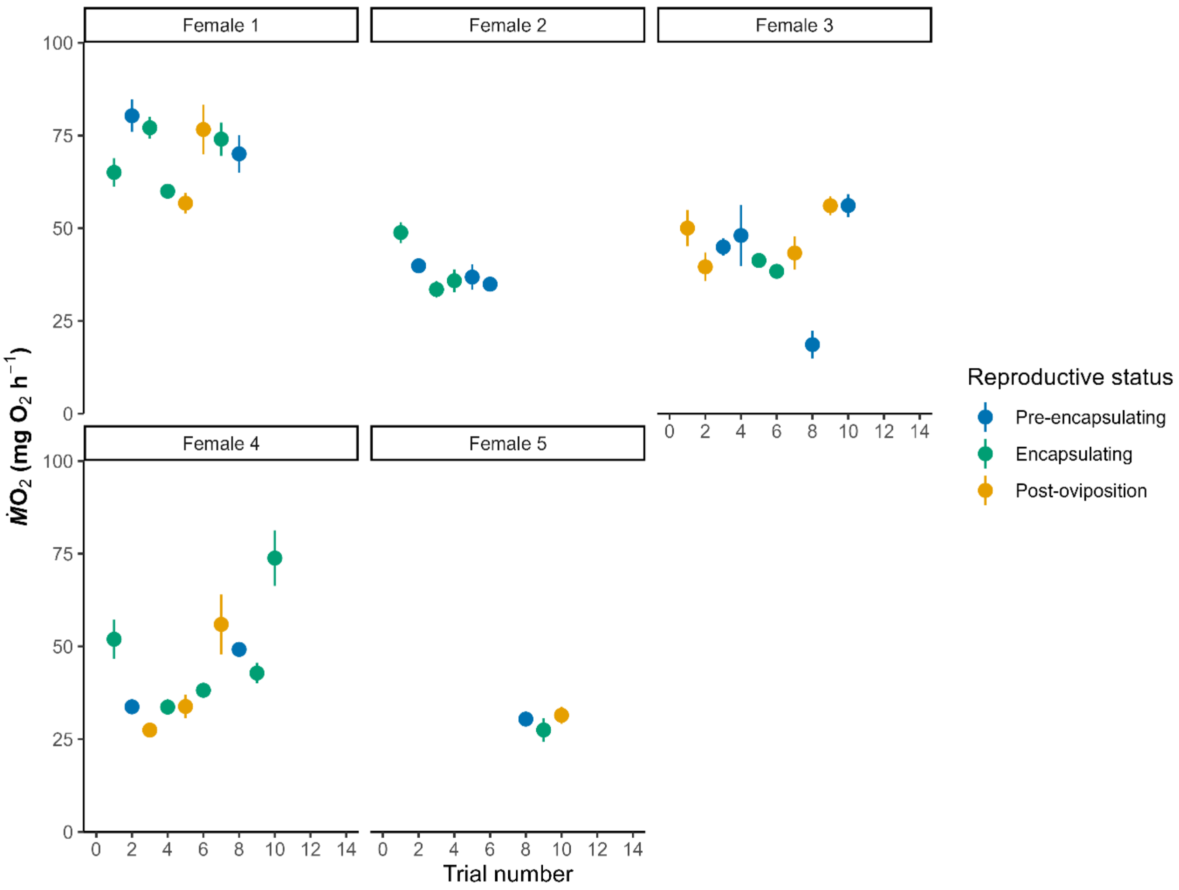


**Fig. S1.** Mean $\dot{M}O2$ (± s.d.) of each trial for each shark (individual panels) in the study by reproductive status represented by colour.

Biology Open • Supplementary information

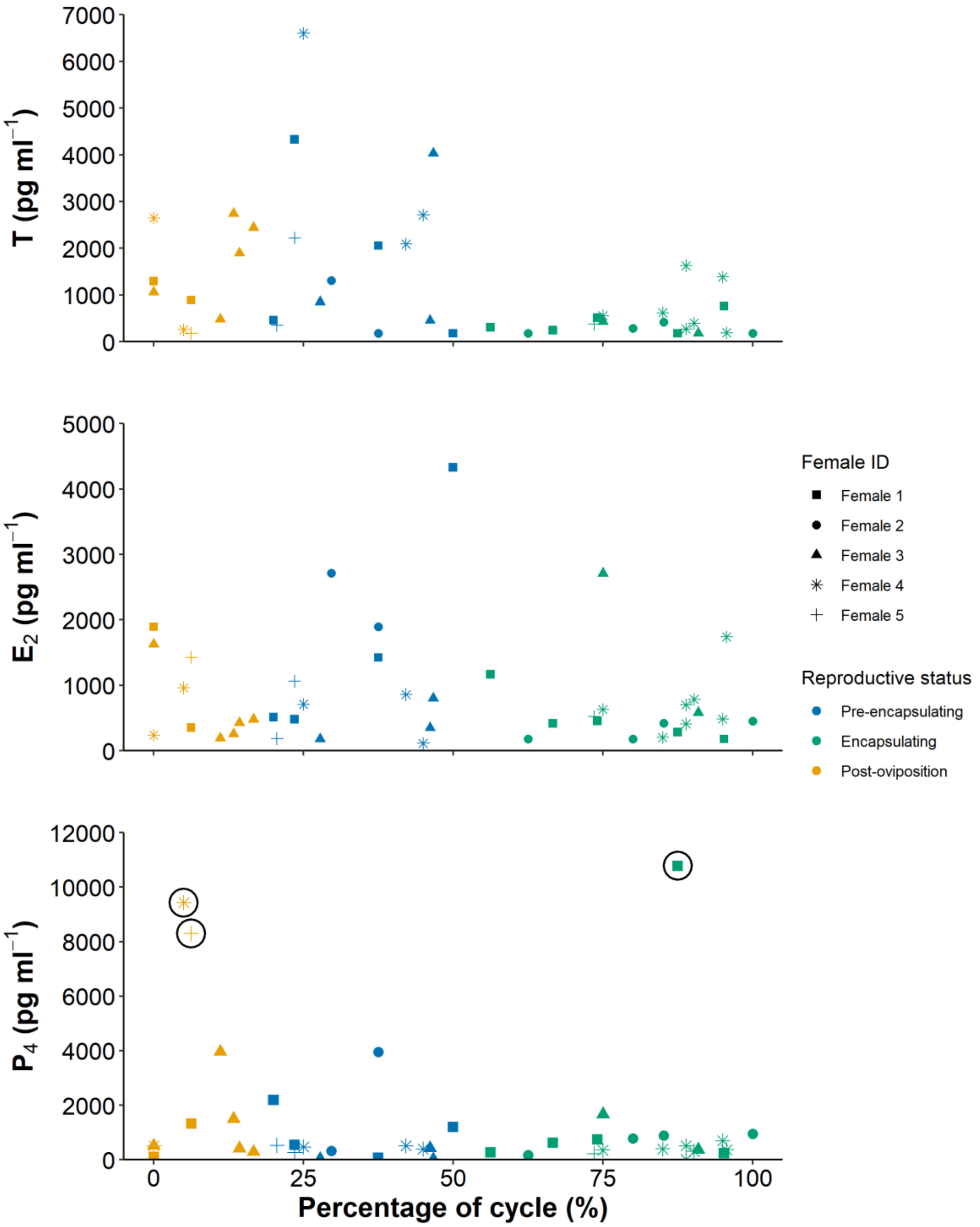


**Fig. S2.** The circulating concentrations of sex steroid hormones (A) testosterone (T (pg ml$^{-1}$)), (B) estradiol ($E_2$ (pg ml$^{-1}$)), and (C) progesterone ($P_4$ (pg ml$^{-1}$)) across an average egg production cycle, where 0 and 100% represent egg deposition of one clutch to the next. Colours represent the three reproductive statuses described in Figure 4.1, and point shapes represent the five female sharks in the study. The circled data points highlight hormonal peaks of note referenced in the main text.

**Table S1.** The mean (± s.d.) water quality parameters monitored hourly (*) and weekly (**) throughout the study.

| Temperature (°C)* | Salinity (ppt)* | pH** | $NH_3$ (ppm)** | Nitrites (ppm)** | Nitrates (ppm) ** |
|---|---|---|---|---|---|
| 24.9 ± 0.28 | 35.2 ± 0.40 | 8.1 ±0.15 | < 0.25 | < 0.25 | < 0.25 |

Biology Open • Supplementary information

**Table S2. $\dot{M}O_2$ across mass:**

A. lmer(log10($\dot{M}O_2$) ~ log(mass) + reproductive status + (1|ID))

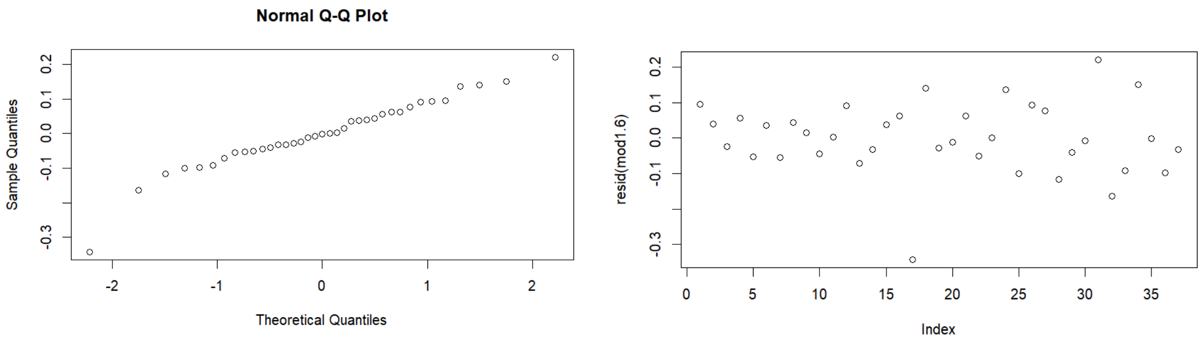


B. Fixed Effects

| Effect Type | Estimate | Std. Error | t-value | Pr(>\|t\|) |
|---|---|---|---|---|
| **(Intercept)** | 1.581 | 0.072 | 21.968 | 3.97e-08 |
| **log10(mass)** | 0.571 | 0.572 | 0.999 | 0.327 |
| **Status: Encapsulating** | 0.023 | 0.044 | 0.525 | 0.604 |
| **Status: Post-oviposition** | 0.025 | 0.050 | 0.498 | 0.622 |

C. Random Effect

| Effect Type | Group | Term | Estimate (SD) |
|---|---|---|---|
| **Random Intercept** | ID | (Intercept) | 0.112 |
| **Residual** | — | Observation-level | 0.111 |

D. Model Fit Statistics

| Statistic | Value |
|---|---|
| **Number of Observations (nobs)** | 37 |
| **Residual Std. Dev. (sigma)** | 0.111 |
| **Log-Likelihood (logLik)** | 19.6 |
| **Akaike Information Criterion (AIC)** | -27.2 |
| **Bayesian Information Criterion (BIC)** | -17.6 |
| **REML Criterion (REMLcrit)** | -39.2 |
| **Degrees of Freedom (Residual)** | 31 |

Biology Open • Supplementary information

## Table S3. Testosterone (T) across the female reproductive cycle

A. aov(log(T) ~ reproductive status + ID)

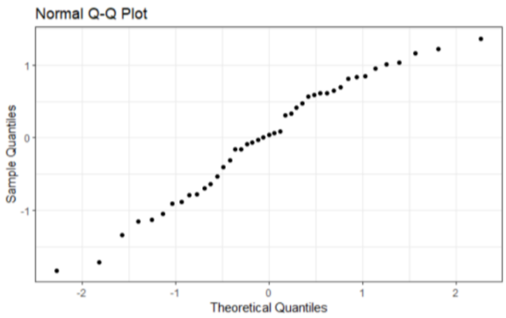


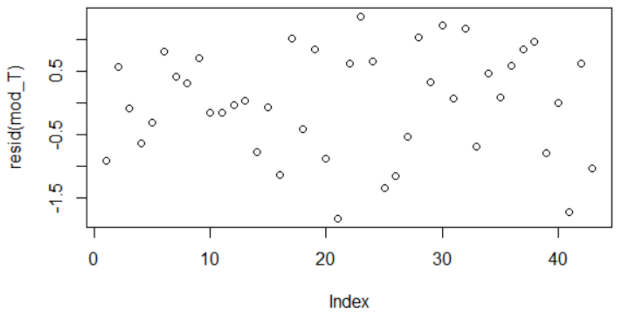


B. ANCOVA Table

| Variable | Sum Sq | df | F value | Pr(>F) |
|---|---|---|---|---|
| **Status** | 11.31359 | 2 | 7.080759 | 0.002551 |
| **ID** | 6.171025 | 4 | 1.931108 | 0.12635 |
| **Residuals** | 28.76028 | 36 | NA | NA |

C. Estimated marginal means

| Status | emmean | SE | df | lower.CL | upper.CL |
|---|---|---|---|---|---|
| **Pre-encapsulating** | 6.987453 | 0.242342 | 36 | 6.495959 | 7.478946 |
| **Encapsulating** | 5.800671 | 0.223294 | 36 | 5.347811 | 6.253532 |
| **Post-oviposition** | 6.675869 | 0.31054 | 36 | 6.046065 | 7.305674 |

**D. Post-hoc comparison**

| Contrast | Estimate | SE | df | t.ratio | p.value |
|---|---|---|---|---|---|
| **(Pre-encapsulating) - Encapsulating** | 1.186782 | 0.323382 | 36 | 3.669908 | 0.0022 |
| **(Pre-encapsulating) - (Post-oviposition)** | 0.311583 | 0.385809 | 36 | 0.807612 | 0.700785 |
| **Encapsulating - (Post-oviposition)** | -0.8752 | 0.383766 | 36 | -2.28055 | 0.071542 |

Biology Open • Supplementary information

**Table S4. 17β-estradiol ($E_2$) across the female reproductive cycle**

A. aov(log($E_2$) ~ reproductive status + ID)

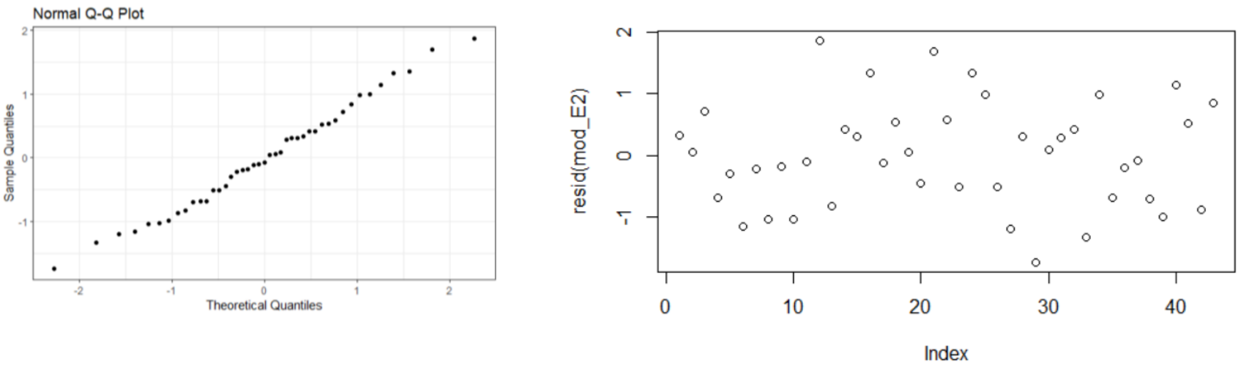


B. ANCOVA Table

| Variable | Sum Sq | df | F value | Pr(>F) |
|---|---|---|---|---|
| **Status** | 0.904292 | 2 | 0.532992 | 0.591406 |
| **ID** | 0.493193 | 4 | 0.145345 | 0.963892 |
| **Residuals** | 30.5394 | 36 | NA | NA |

C. Estimated marginal means

| Status | emmean | SE | df | lower.CL | upper.CL |
|---|---|---|---|---|---|
| **Pre-encapsulating** | 6.527483 | 0.249726 | 36 | 6.021016 | 7.03395 |
| **Encapsulating** | 6.185949 | 0.230097 | 36 | 5.719291 | 6.652606 |
| **Post-oviposition** | 6.399012 | 0.320001 | 36 | 5.750019 | 7.048004 |

Biology Open • Supplementary information

## Table S5. Progesterone ($P_4$) across the female reproductive cycle

A. aov(log($P_4$) ~ reproductive status + ID)

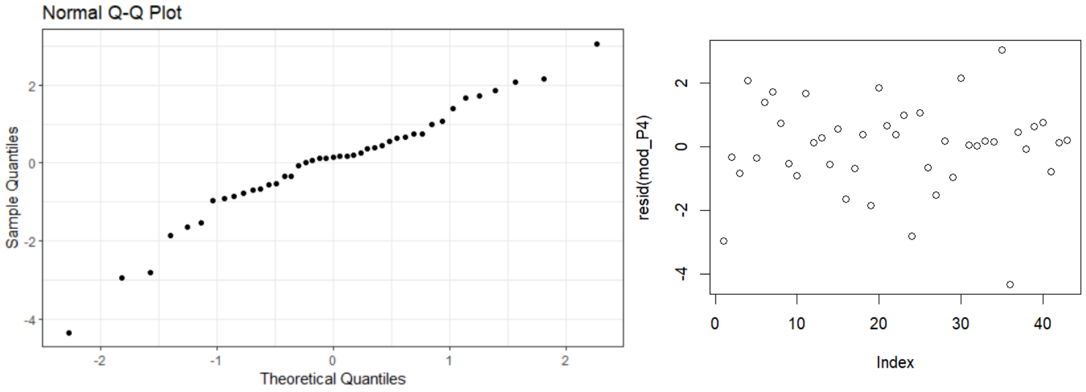


B. ANCOVA Table

| Variable | Sum Sq | df | F value | Pr(>F) |
|---|---|---|---|---|
| **Status** | 13.13154 | 2 | 2.850299 | 0.070939 |
| **ID** | 8.310589 | 4 | 0.901937 | 0.47308 |
| **Residuals** | 82.92738 | 36 | NA | NA |

C. Estimated marginal means

| Status | emmean | SE | df | lower.CL | upper.CL |
|---|---|---|---|---|---|
| **Pre-encapsulating** | 5.85005 | 0.411511 | 36 | 5.015467 | 6.684634 |
| **Encapsulating** | 6.058091 | 0.379165 | 36 | 5.289108 | 6.827074 |
| **Post-oviposition** | 7.353287 | 0.527315 | 36 | 6.283842 | 8.422731 |

Biology Open • Supplementary information

**Table S6. Hct across the female reproductive cycle**

A. aov(log(hct) ~ reproductive status + ID)

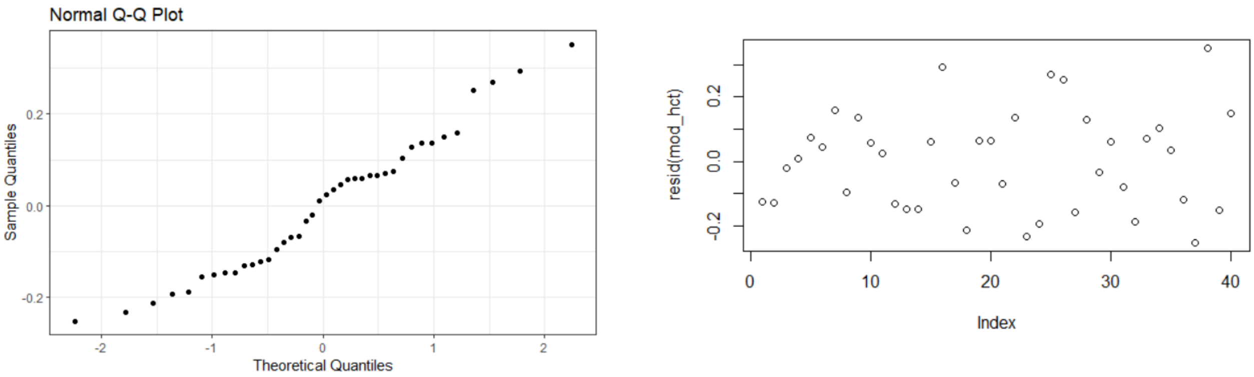


B. ANCOVA Table

| Variable | Sum Sq | df | F value | Pr(>F) |
|---|---|---|---|---|
| **Status** | 0.028451 | 2 | 0.516587 | 0.601297 |
| **ID** | 0.795315 | 4 | 7.220301 | 2.72E-04 |
| **Residuals** | 0.908736 | 33 | NA | NA |

C. Estimated marginal means

| Status | emmean | SE | df | lower.CL | upper.CL |
|---|---|---|---|---|---|
| **Pre-encapsulating** | 2.666355 | 0.04327 | 33 | 2.578321 | 2.75439 |
| **Encapsulating** | 2.670282 | 0.059668 | 33 | 2.548885 | 2.791678 |
| **Post-oviposition** | 2.611347 | 0.045029 | 33 | 2.519736 | 2.702958 |

Biology Open • Supplementary information

**Table S7. Hb across the female reproductive cycle**

A. aov(log(hb) ~ reproductive status + ID)

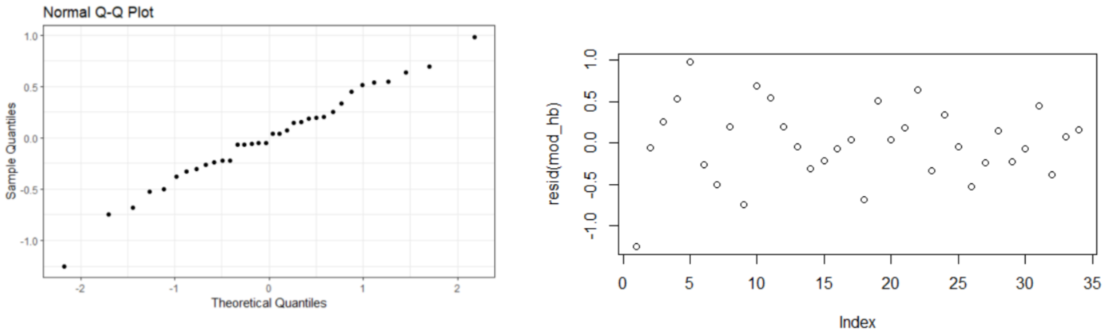


B. ANCOVA Table

| Variable | Sum Sq | df | F value | Pr(>F) |
|---|---|---|---|---|
| **Status** | 0.276393 | 2 | 0.537493 | 0.590333 |
| **ID** | 0.131469 | 4 | 0.127832 | 0.971028 |
| **Residuals** | 6.942062 | 27 | NA | NA |

C. Estimated marginal means

| Status | emmean | SE | df | lower.CL | upper.CL |
|---|---|---|---|---|---|
| **Pre-encapsulating** | 3.580669 | 0.140706 | 27 | 3.291964 | 3.869373 |
| **Encapsulating** | 3.471021 | 0.216311 | 27 | 3.027187 | 3.914855 |
| **Post-oviposition** | 3.719474 | 0.141931 | 27 | 3.428255 | 4.010693 |

Biology Open • Supplementary information

**Table S8. MCHC across the female reproductive cycle**

D. aov(log(MCHC) ~ reproductive status + ID)

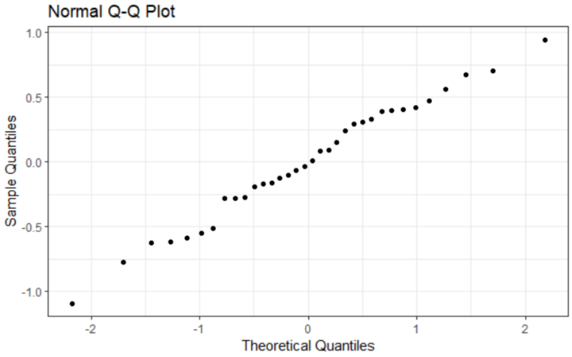


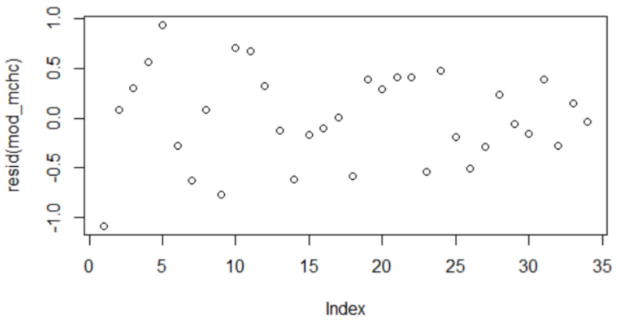


E. ANCOVA Table

| Variable | Sum Sq | df | F value | Pr(>F) |
|---|---|---|---|---|
| **Status** | 0.364101 | 2 | 0.679151 | 0.515499 |
| **ID** | 0.642732 | 4 | 0.599438 | 0.666216 |
| **Residuals** | 7.237512 | 27 | NA | NA |

F. Estimated marginal means

| Status | emmean | SE | df | lower.CL | upper.CL |
|---|---|---|---|---|---|
| **Pre-encapsulating** | 5.500174 | 0.143669 | 27 | 5.205389 | 5.794958 |
| **Encapsulating** | 5.445094 | 0.220866 | 27 | 4.991914 | 5.898275 |
| **Post-oviposition** | 5.696262 | 0.14492 | 27 | 5.398911 | 5.993614 |

Biology Open • Supplementary information
